# Sulfur Mitigates Cadmium Toxicity in Lettuce via Phytochelatins and the AsA–GSH Cycle

**DOI:** 10.1021/acs.jafc.5c09833

**Published:** 2025-10-13

**Authors:** Kuei−San Chen, Hung−Yu Lai

**Affiliations:** Department of Soil and Environmental Sciences, 34916 National Chung Hsing University, Taichung 402202, Taiwan

**Keywords:** AsA−GSH cycle, cadmium, lettuce, phytochelatins, sulfur

## Abstract

Cadmium (Cd), a toxic heavy metal, accumulates in crops and poses health risks. Sulfur (S) enhances plant metabolism and tolerance to heavy metal stress. In this hydroponic study, we investigated the effects of S on lettuce growth, sulfur-containing compounds, and Cd uptake under Cd stress. S promoted growth, reduced Cd accumulation, and increased phytochelatins (PCs) and PCs–Cd complexes, limiting Cd translocation. Subcellular analysis suggested decreased Cd in organelles and enhanced sequestration in vacuoles and the cell wall, while S alleviated oxidative damage via the AsA–GSH cycle. These findings, obtained under hydroponic conditions, may differ from those of field environments and require further validation. This study is novel in directly quantifying PCs–Cd complexes and analyzing subcellular Cd distribution, providing environmentally relevant insights for strategies to reduce Cd accumulation in crops and improve food safety.

## Graphical abstract

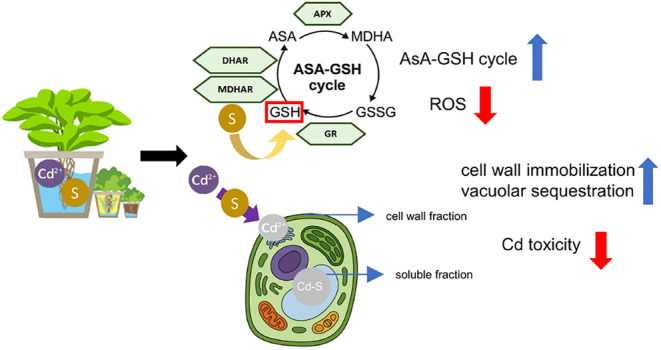

## 1 Introduction

Due to cadmium (Cd) high mobility and toxicity, it can accumulate in edible plant tissues and subsequently be introduced into the food chain, raising public health concerns.^1–3^ Cd, once absorbed by roots, is distributed throughout plant tissues, where it disrupts physiological activities by inducing excessive reactive oxygen species (ROS) generation, leading to oxidative stress, lipid peroxidation, cellular membrane damage, and visible symptoms such as root necrosis, leaf yellowing and curling, ultimately reducing biomass, suppressing growth, and lowering yield.^4–8^

Before entering plant cells, heavy metals (HMs) are first adsorbed onto the root surface and bind to cell wall components, such as the carboxyl groups in aldehydic acids or polysaccharides.^9–12^ Although the cell wall acts as a protective barrier by immobilizing HMs and mitigating their toxicity, some of these metals can still penetrate the cell. To counteract the damage caused by HMs, plants have evolved detoxification strategies that involve producing sulfur-containing compounds, such as glutathione (GSH), phytochelatins (PCs), and metallothioneins (MTs). These compounds bind to HMs, forming complexes that are transported into vacuoles, thereby decreasing mobility and toxicity of HMs.^13^ Besides the immobilization of HMs in the cell wall, vacuolar sequestration represents a crucial detoxification pathway in plants. The vacuole serves as a major reservoir for various ions and metabolites and plays a vital role in both detoxification processes and maintaining cellular homeostasis.^14^

Sulfur (S) is a vital nutrient necessary for plant growth and development, playing an important role in enhancing plant tolerance to HMs.^15^ Inside cells, S assimilation involves key enzymes for cysteine (Cys) biosynthesis. Through various enzyme-catalyzed pathways, Cys contributes to the formation of important sulfur-containing compounds.^16^ In plants, sulfur-containing compounds—specifically GSH, PCs, and MTs—are known to facilitate Cd detoxification through chelation mechanisms.^17^

Numerous studies have demonstrated that S reduced Cd uptake and translocation in plants by boosting the synthesis of sulfur-containing compounds, such as GSH and PCs, thereby alleviating Cd toxicity. This effect has been observed in various crops including rice,^18^ wheat,^19^ and Chinese cabbage.^20,21^ Moreover, S enhanced the ascorbate–glutathione (AsA–GSH) cycle, playing a vital role in mitigating HMs-induced oxidative stress. For example, in hydroponically grown Chinese cabbage and tartary buckwheat exposed to Cd, S application alleviated oxidative damage mainly by activating the AsA–GSH cycle.^22,23^

Lettuce (*Lactuca sativa* L.) is a widely consumed leafy vegetable, particularly popular in Asian diets,^24^ and in 2023, approximately 195,000 ha of farmland in the United States was dedicated to its cultivation, highlighting its significance as a major leafy vegetable crop.^25^ Its rapid growth and higher capacity to accumulate Cd compared with other leafy vegetables make lettuce a useful test plant for studies of Cd-contaminated soils.^26,27^ While S supplementation has been shown to alleviate heavy metal stress in plants, the precise mechanisms through which S reduces Cd toxicity—particularly in lettuce, remain poorly understood. Specifically, there is limited information about (i) how Cd is detoxified in lettuce roots and shoots, (ii) the interaction between S metabolism and Cd accumulation, and (iii) the effect of different growth stages on Cd translocation and oxidative stress responses in lettuce. Unlike previous studies that mainly focused on cereals or *Brassicaceae* crops, this work provides novel insights into lettuce as a Cd-accumulating leafy vegetable. By integrating growth-stage-dependent responses, thiol metabolism, and the AsA–GSH cycle, this study advances the understanding of how S mitigates Cd toxicity in lettuce, thereby filling a critical knowledge gap in vegetable crops.

## 2 Materials and Methods

### 2.1 Plant Material and Growth Conditions

Lettuce seeds (*Lactuca sativa* L. cv. Sweet) were disinfected using a 2% (v/v) sodium hypochlorite solution for 5 min. After sterilization, seeds were placed on moistened filter paper to germinate at ambient temperature for 48 h. Seedlings that germinated uniformly were chosen and transplanted into a Hoagland nutrient solution for treatment. Each treatment was conducted using plastic pots, with three replicates and 12 plants per pot. To establish different experimental conditions, cadmium chloride and/or sodium sulfate were added to the nutrient solution as follows: (1) CK, 0 μM CdCl_2_ + 0 mM Na_2_SO_4_; (2) Cd, 40 μM CdCl_2_ + 0 mM Na_2_SO_4_; (3) CdS, 40 μM CdCl_2_ + 4 mM Na_2_SO_4_. The solution was maintained at pH 6.5 and replaced every 3 days. All treatments were carried out in a growth chamber with controlled conditions: 14 h light/10 h dark cycle, temperature of 25.0 ± 1.5 °C, and relative humidity of 60.0 ± 2.4%. Plant samples were collected on days 28, 42, and 56 after sowing (denoted as days D28, D42, and D56, respectively).

### 2.2 Plant Sampling

The relative chlorophyll content was first determined by using a SPAD-502 Plus chlorophyll meter (Konica Minolta, Osaka, Japan). Subsequently, the plant height and root length were measured. To eliminate HMs bound to the root surface, roots were immersed in 20 mM disodium ethylenediaminetetraacetic acid (Na_2_-EDTA) solution for 15 min. After cleaning, the plants were split into two portions. One portion was oven-dried at 70 °C for a minimum of 72 h, weighed to determine dry biomass, ground into powder, and used to analyze Cd and S contents. The other portion was rapidly frozen at −80 °C and later used for biochemical assays, including H_2_O_2_ content, Cd subcellular distribution, thiol compound content, PCs and PCs–Cd complex, as well as related enzyme activities involved in the AsA–GSH cycle. Each analysis was performed in triplicate to ensure measurement reliability, and the reported values represent the mean of these replicates.

### 2.3 Analysis of Cd and S in the Root and Shoot

About 0.2 g of finely ground, oven-dried root and shoot samples were subjected to acid digestion using a nitric acid and perchloric acid mixture, following the procedure outlined by Jones and Case.^28^ The resulting digests were filtered, and the concentrations of total Cd and S were quantified by using inductively coupled plasma mass spectrometry (ICP–MS; Elan DRC II, PerkinElmer, Waltham, MA) and ion chromatography (IC; 930 Compact IC Flex, Metrohm, Herisau, Switzerland), respectively.

### 2.4 Subcellular Distribution of Cd

Subcellular distribution of fresh root and shoot tissues was performed following the method described by Xin et al.,^29^ with slight modifications. Fresh samples were ground in a prechilled mortar with an ice-cold extraction buffer containing 250 mM sucrose, 1 mM dithiothreitol (DTT), and 50 mM Tris–HCl (pH 7.5). The homogenate was subjected to sequential centrifugation at 4 °C to isolate different cellular components. The first spin at 300*g* for 30 s yielded a pellet corresponding to the cell wall fraction (F_CW_). The supernatant was then centrifuged at 2000*g* for 15 min to obtain the nuclear and chloroplast fraction (F_NC_), followed by a 10,000*g* spin for 20 min to isolate the mitochondrial fraction (F_M_). The final supernatant represented the soluble fraction (F_S_). Each subcellular fraction was digested with a HNO_3_/HClO_4_ mixture, and Cd concentrations were measured by using ICP–MS.

### 2.5 Analysis of Thiol and PCs–Cd in the Root and Shoot

Thiol compounds analyzed in this study included Cys, GSH, phytochelatin 2 (PC_2_), phytochelatin 3 (PC_3_), and phytochelatin 4 (PC_4_). For extraction, fresh plant tissues were homogenized in 2 mL of extraction buffer containing 0.1% trifluoroacetic acid (TFA) and 6.3 mM diethylenetriaminepentaacetic acid (DTPA), supplemented with 1 mL of tris­(2-carboxyethyl) phosphine hydrochloride (TCEP). The homogenates were centrifuged at 12,000*g* for 20 min at 4 °C, following the procedure reported by Wu et al.^30^ Sample derivatization was carried out according to the method of Sneller et al.,^31^ and thiol content was determined using high-performance liquid chromatography (HPLC; CM5000, HITACHI, Tokyo, Japan).

PCs–Cd complexes in fresh root and shoot tissues were analyzed based on protocols described by Chen et al.,^32^ Miszczak et al.,^33^ and Navaza et al.^34^ Approximately 2 g of fresh tissue was extracted using 2 mL of 25 mM ammonium acetate buffer (pH 7.8), followed by ultrasonic treatment in a water bath for 30 min. After centrifugation (12,000*g*, 20 min), the supernatant was filtered through a 0.22 μm membrane. Separation of PCs and PCs–Cd complexes was achieved using a size exclusion chromatography column (Superdex Peptide HR 10/300 GL), and analysis was performed using SEC–HPLC–UV (at 280 nm) coupled with quadrupole time-of-flight mass spectrometry (Q–TOF; G6545B, Agilent Technologies, Santa Clara).

### 2.6 Analysis of H_2_O_2_ and AsA in the Root and Shoot

To determine H_2_O_2_ content, a colorimetric assay adapted from Jana and Choudhuri^35^ was used. Fresh plant material (2 g) was extracted with 3 mL of phosphate buffer (pH 6.5) under chilled conditions (4 °C). After centrifugation at 6000*g* for 25 min, the supernatant was collected and adjusted to 3 mL, using the same buffer. A 3 mL aliquot of this extract was then mixed with 1 mL of 0.1% titanium sulfate in 20% sulfuric acid. Following a second centrifugation at 6000*g* for 15 min, the absorbance of the supernatant was measured at 410 nm. H_2_O_2_ content was determined using a calibration curve prepared with standard H_2_O_2_ solutions.

A modified colorimetric assay based on Liang et al.^22^ was employed to determine the total AsA content. Briefly, 0.2 g of fresh tissue was extracted using ice-cold 5% (w/v) metaphosphoric acid and centrifuged at 22,000*g* for 15 min at 4 °C. To initiate the analysis, 0.3 mL of the supernatant was mixed with 0.75 mL of 150 mM phosphate buffer (pH 7.4) supplemented with 5 mM EDTA, along with 0.15 mL of 10 mM DTT. Following a 10 min incubation at 25 °C to reduce dehydroascorbic acid, 0.15 mL of 0.5% N-ethylmaleimide was added to neutralize excess DTT. The chromogenic reaction was initiated by the sequential addition of 0.6 mL of 10% TCA, 44% ortho-phosphoric acid, 4% α,α′-dipyridyl (dissolved in 70% ethanol), and 0.3% FeCl_3_. After mixing, the solution was incubated at 40 °C for 40 min, and absorbance was recorded at 525 nm. Quantification was conducted using a standard calibration curve of known AsA concentrations.

### 2.7 Enzyme Activity Assays of the AsA–GSH Cycle

Fresh plant tissues (2 g) were ground in liquid nitrogen and extracted with 0.8 mL of potassium phosphate buffer (50 mM, pH 7.0) containing 0.1 mM EDTA. The homogenate was centrifuged at 12,000*g* for 5 min at 4 °C, and the supernatant was collected and used as the crude enzyme extract. Ascorbate peroxidase (APX) activity was assessed following the method of Nakano and Asada^36^ by tracking the decline in absorbance at 290 nm due to ascorbate oxidation. A total of 100 μL of enzyme extract was added to 800 μL of a reaction mixture containing 50 mM potassium phosphate buffer (pH 7.0), 0.5 mM ascorbate, and 0.1 mM EDTA. After stabilization of baseline absorbance at 290 nm, the reaction was initiated by adding 100 μL of H_2_O_2_, and the decrease in absorbance was recorded over 5 min to determine APX activity. Glutathione reductase (GR) activity was measured based on the method by Goldberg and Spooner^37^ by monitoring the decrease in absorbance at 340 nm corresponding to NADPH oxidation. An 800 μL reaction mixture containing 100 mM potassium phosphate buffer (pH 7.8), 2 mM EDTA, and 2.2 mM oxidized glutathione (GSSG) was mixed with 100 μL of enzyme extract. Once the absorbance at 340 nm stabilized, the reaction was started by adding NADPH to reach a final concentration of 0.15 mM. The decline in absorbance at 340 nm was measured for 5 min to calculate GR activity. The activity of dehydroascorbate reductase (DHAR) was assessed by measuring the increase in absorbance at 265 nm, which reflects the formation of ascorbate, following the method of Nakano and Asada.^36^ The reaction system (900 μL) contained 50 mM potassium phosphate buffer (pH 7.0), 0.1 mM EDTA, 2.5 mM reduced glutathione (GSH), and 1 mg mL^–1^ dehydroascorbate (DHA). A 100 μL aliquot of enzyme extract was added to initiate the reaction, and the increase in absorbance at 265 nm was recorded for 5 min to estimate DHAR activity.

### 2.8 Statistical Analysis

All statistical analyses were performed using R software (version 4.4.3), and figures were generated using SigmaPlot version 14.0. Data were first checked for normality and homogeneity of variance. Treatment differences were then assessed by one-way analysis of variance (ANOVA), followed by least significant difference (LSD) tests for multiple comparisons. Statistical significance was set at *p* < 0.05.

## 3 Results

### 3.1 Biomass, Relative Chlorophyll Content, and Oxidative Stress of Lettuce

As illustrated in Figure 1a, exposure to 40 μM Cd significantly (*p* < 0.05) reduced both shoot height and root length in lettuce compared to the control. Specifically, shoot height declined by 58.3–64.7%, and root length decreased by 44.4–60.0% on D28, D42, and D56, respectively. The addition of S markedly improved both parameters under Cd stress, showing significant (*p* < 0.05) increases in shoot height and root length compared to S-deficient plants subjected to the same Cd treatment at each time point. Under Cd stress, shoot and root dry weights were also significantly reduced (*p* < 0.05) relative to the control group (Figure 1b). However, S supplementation notably alleviated these reductions, significantly enhancing shoot and root biomass across all three sampling dates compared to those with Cd-only treatments. Relatively chlorophyll content, indicated by SPAD readings, also declined significantly (*p* < 0.05) under Cd stress (Figure 2a). In contrast, S application led to significant (*p* < 0.05) increases in SPAD readings in Cd-treated plants at all time points when compared to the Cd-only treatments. Furthermore, Cd exposure led to a noticeable rise in H_2_O_2_ content in lettuce shoots compared to the control (Figure 2b). The presence of S significantly (*p* < 0.05) mitigated this oxidative stress, as shown by reduced H_2_O_2_ content in Cd-stressed plants receiving S supplementation. Throughout the experimental period, plant height, root length, and dry weight peaked at D42, followed by either a plateau or a slight decline by D56.

**Figure 1:**
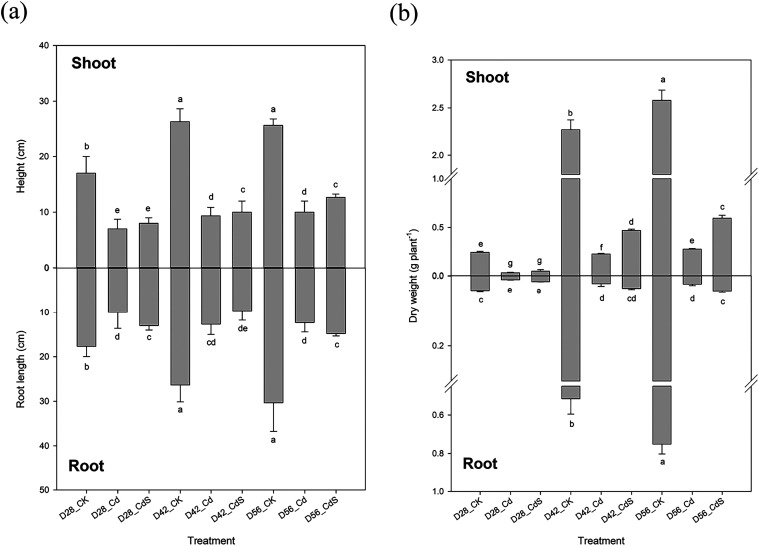
Root length, shoot height (a), and dry weight (b) of the lettuce after the hydroponic experiment under different treatments. Small letter indicates statistical differences among treatments (LSD test, *p* < 0.05). CK: 0 μM CdCl_2_ + 0 mM Na_2_SO_4_; Cd: 40 μM CdCl_2_ + 0 mM Na_2_SO_4_; CdS: 40 μM CdCl_2_ + 4 mM Na_2_SO_4_; D28: harvest at 28 days; D42: harvest at 42 days; D56: harvest at 56 days.

**Figure 2:**
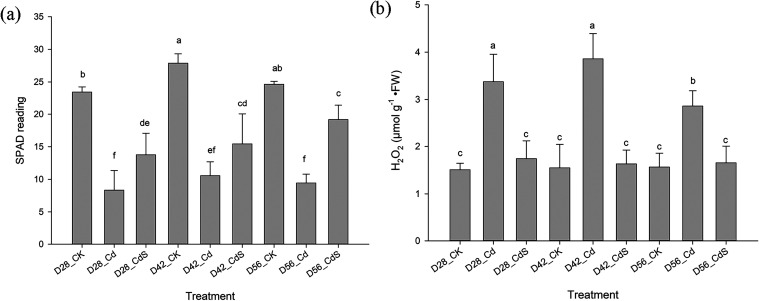
SPAD reading (a) and H_2_O_2_ (b) concentration of the lettuce in shoots after the hydroponic experiment under different treatments. Small letter indicates statistical differences among treatments (LSD test, *p* < 0.05). CK: 0 μM CdCl_2_ + 0 mM Na_2_SO_4_; Cd: 40 μM CdCl_2_ + 0 mM Na_2_SO_4_; CdS: 40 μM CdCl_2_ + 4 mM Na_2_SO_4_; D28: harvest at 28 days; D42: harvest at 42 days; D56: harvest at 56 days.

### 3.2 Concentration of Cd and S

Under Cd stress, Cd accumulation in lettuce tissues increased markedly over time. On D28, D42, and D56, Cd concentrations in the shoots reached 30, 535, and 406 mg kg^–1^, while the corresponding values in the roots were 261, 732, and 747 mg kg^–1^, respectively (Figure 3a). Compared with the Cd-only treatment, the addition of S significantly reduced Cd content in both plant parts. Under Cd treatment, S concentrations in the shoots were 0.80, 0.58, and 0.47%, while those in the roots were 1.80, 1.85, and 1.08% on D28, D42, and D56, respectively (Figure 3b). Supplementation with S led to a significant (*p* < 0.05) increase in S concentrations in both shoots and roots compared to plants subjected to Cd stress alone. Cd mobility within the plant was assessed by calculating the translocation factor (TF), which is expressed as the ratio of Cd concentration in the shoots to that in the roots. Under Cd stress, TF values were 0.87, 0.73, and 0.54 on D28, D42, and D56, respectively (Figure S1). S treatment under Cd stress further reduced the TF values, indicating a decrease in Cd translocation from roots to shoots.

**Figure 3:**
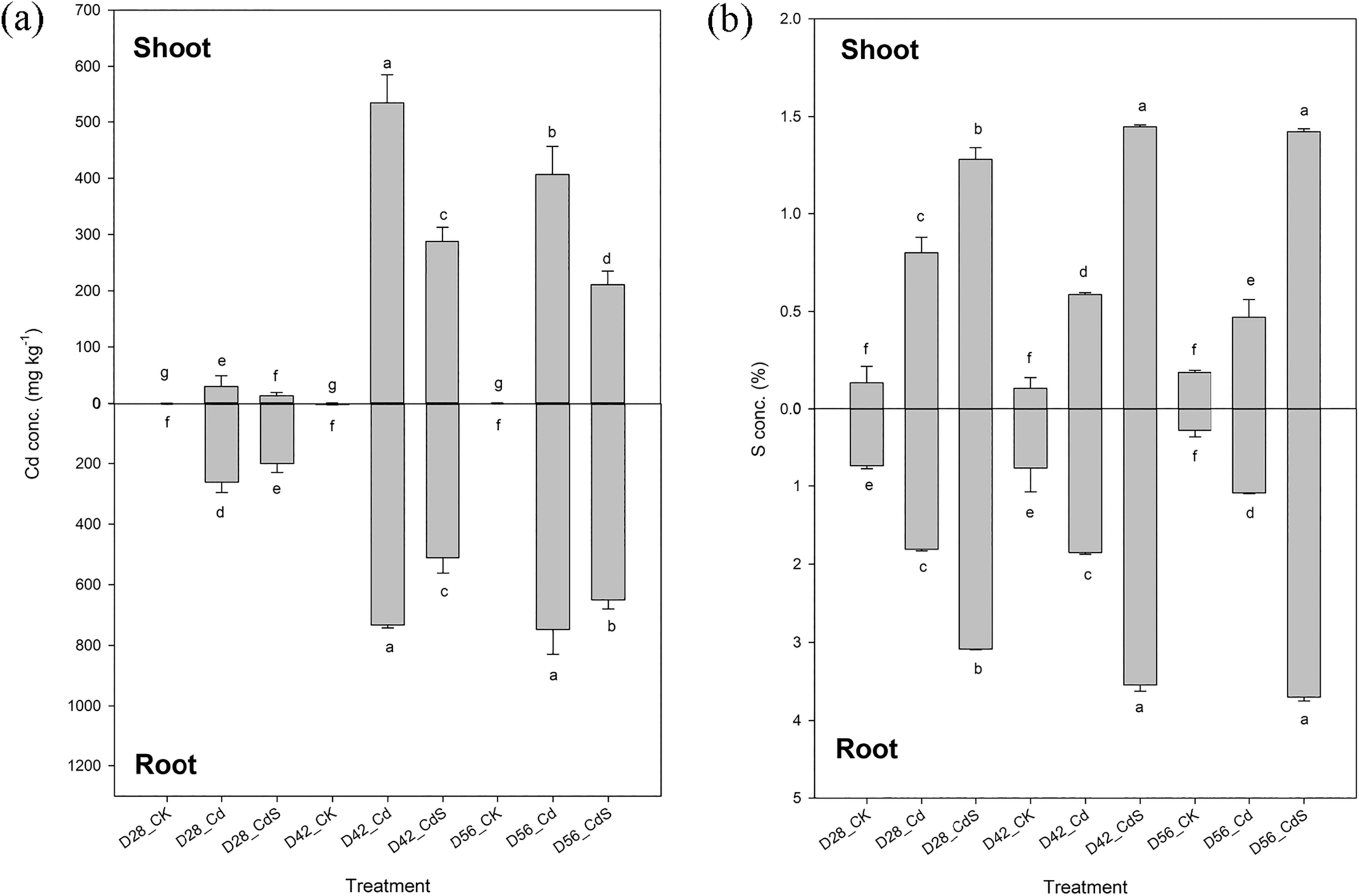
Cd (a) and S (b) concentrations of lettuce after the hydroponic experiment under different treatments. Small letter indicates statistical differences among treatments (LSD test, *p* < 0.05). CK: 0 μM CdCl_2_ + 0 mM Na_2_SO_4_; Cd: 40 μM CdCl_2_ + 0 mM Na_2_SO_4_; CdS: 40 μM CdCl_2_ + 4 mM Na_2_SO_4_; D28: harvest at 28 days; D42: harvest at 42 days; D56: harvest at 56 days.

### 3.3 Subcellular Distribution of Cd

The subcellular distribution of Cd in lettuce shoots and roots is presented in Figure 4. Among the various fractions, the F_S_ fraction consistently exhibited the highest proportion of Cd accumulation in both tissues. In shoots, Cd was predominantly found in the F_S_ fraction, accounting for 86–93% of total Cd, which was significantly higher than the proportions in the F_CW_, F_NC_, and F_M_ under Cd exposure. Similarly, in roots, the F_S_ fraction held the majority of Cd (58–88%), surpassing F_CW_, F_NC_, and F_M_ fractions. Notably, S application altered the subcellular distribution of Cd. On D28 and D42, Cd in both shoots and roots shifted from the F_NC_ and F_M_ fractions toward the F_S_ fraction, whereas by D56, Cd distribution shifted toward the F_CW_ fraction, suggesting a time-dependent modification of Cd sequestration under S supplementation. It should be noted that subcellular fractionation provides enrichment rather than precise organelle localization; however, these results are consistent with previous TEM observations^38^ showing Cd accumulation in the cell wall and other compartments, supporting the reliability of our interpretations.

**Figure 4:**
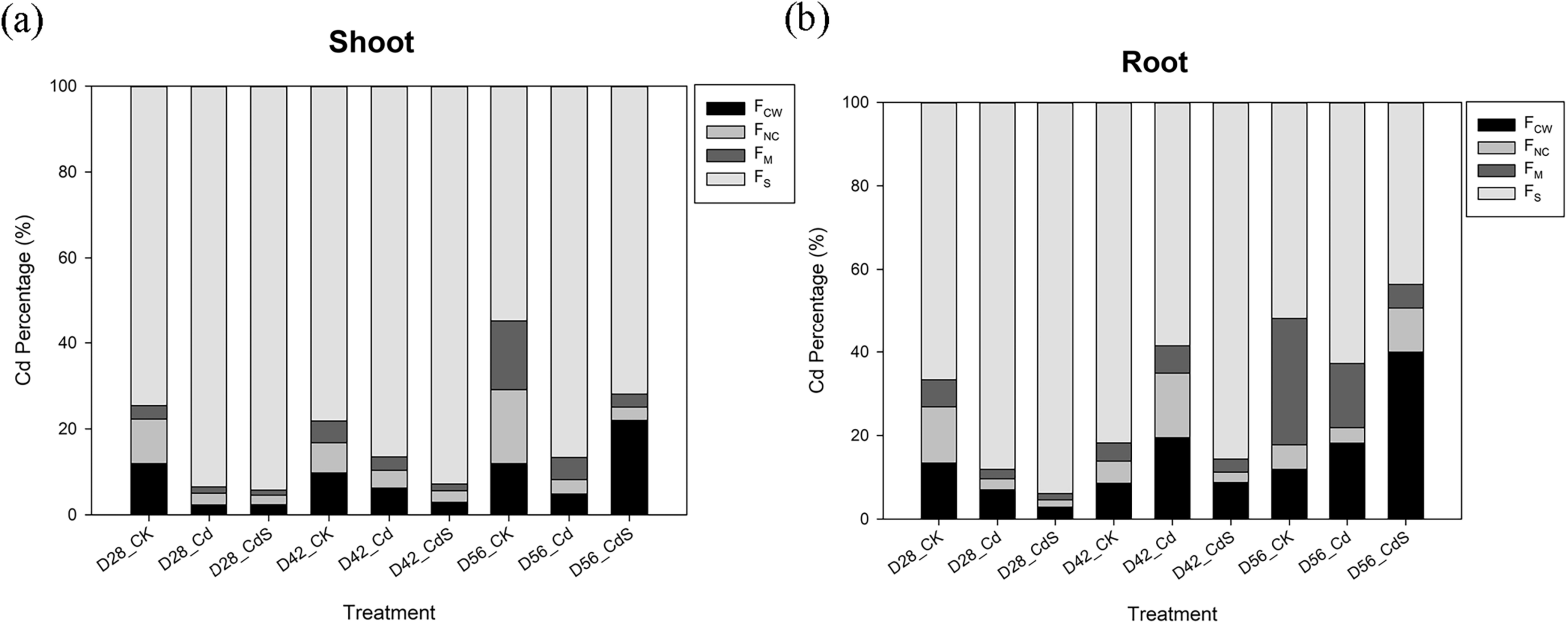
Subcellular fractions of Cd in the shoots (a) and roots (b) of lettuce after the hydroponic experiment under different treatments. CK: 0 μM CdCl_2_ + 0 mM Na_2_SO_4_; Cd: 40 μM CdCl_2_ + 0 mM Na_2_SO_4_; CdS: 40 μM CdCl_2_ + 4 mM Na_2_SO_4_; D28: harvest at 28 days; D42: harvest at 42 days; D56: harvest at 56 days. F_CW_: cell wall fraction; F_NC_: nuclear and chloroplast fraction; F_M_: mitochondrial fraction; F_S_: soluble fraction.

### 3.4 Analysis of Thiol, PCs, and PCs–Cd Complexes

As illustrated in Figure 5, the thiol content in lettuce shoots exposed to Cd reached 621, 840, and 900 nmol g^–1^ on D28, D42, and D56, respectively, while root concentrations were 698, 3710, and 3559 nmol g^–1^. The addition of exogenous S significantly (*p* < 0.05) elevated thiol content in comparison to Cd treatment alone. Thiol content in both tissues reached a maximum on D42, followed by a slight decline by D56. Figure 6 presents the proportion of various thiol compounds in shoots and roots, including Cys, GSH, PC_2_, PC_3_, and PC_4_. Among these, GSH consistently represented the largest fraction, constituting 76–92% in shoots and 65–94% in roots across all treatments. S supplementation notably increased the proportion of PCs relative to both the control and Cd-only treatments. Tables 1 and 2 show the distributions of PCs and PCs–Cd complexes in shoots and roots. Under Cd treatment, PCs–Cd complexes comprised 21.43, 27.31, and 60.18% of total PCs in shoots on D28, D42, and D56, respectively, while in roots, these percentages were 32.35, 45.25, and 87.22%. Interestingly, PC_2_–Cd was dominant in shoots on D28, whereas PC_4_–Cd became the dominant form under all of the other conditions. Exogenous S further boosted the proportion of PCs–Cd complexes compared to Cd treatment alone.

**Figure 5:**
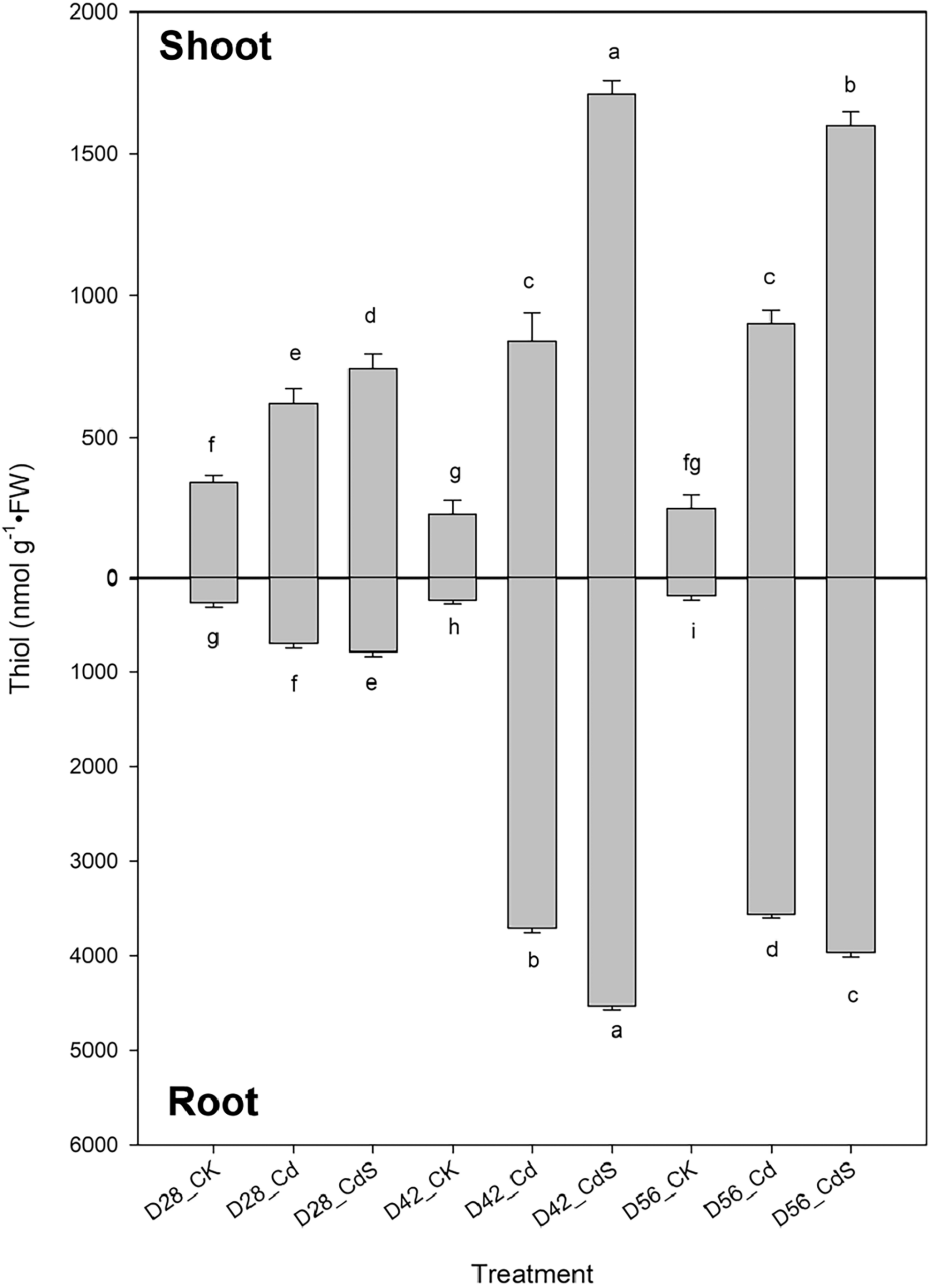
Total amount of thiols after the hydroponic experiment under different treatments. Small letter indicates statistical differences among treatments (LSD test, *p* < 0.05). CK: 0 μM CdCl_2_ + 0 mM Na_2_SO_4_; Cd: 40 μM CdCl_2_ + 0 mM Na_2_SO_4_; CdS: 40 μM CdCl_2_ + 4 mM Na_2_SO_4_; D28: harvest at 28 days; D42: harvest at 42 days; D56: harvest at 56 days.

**Figure 6:**
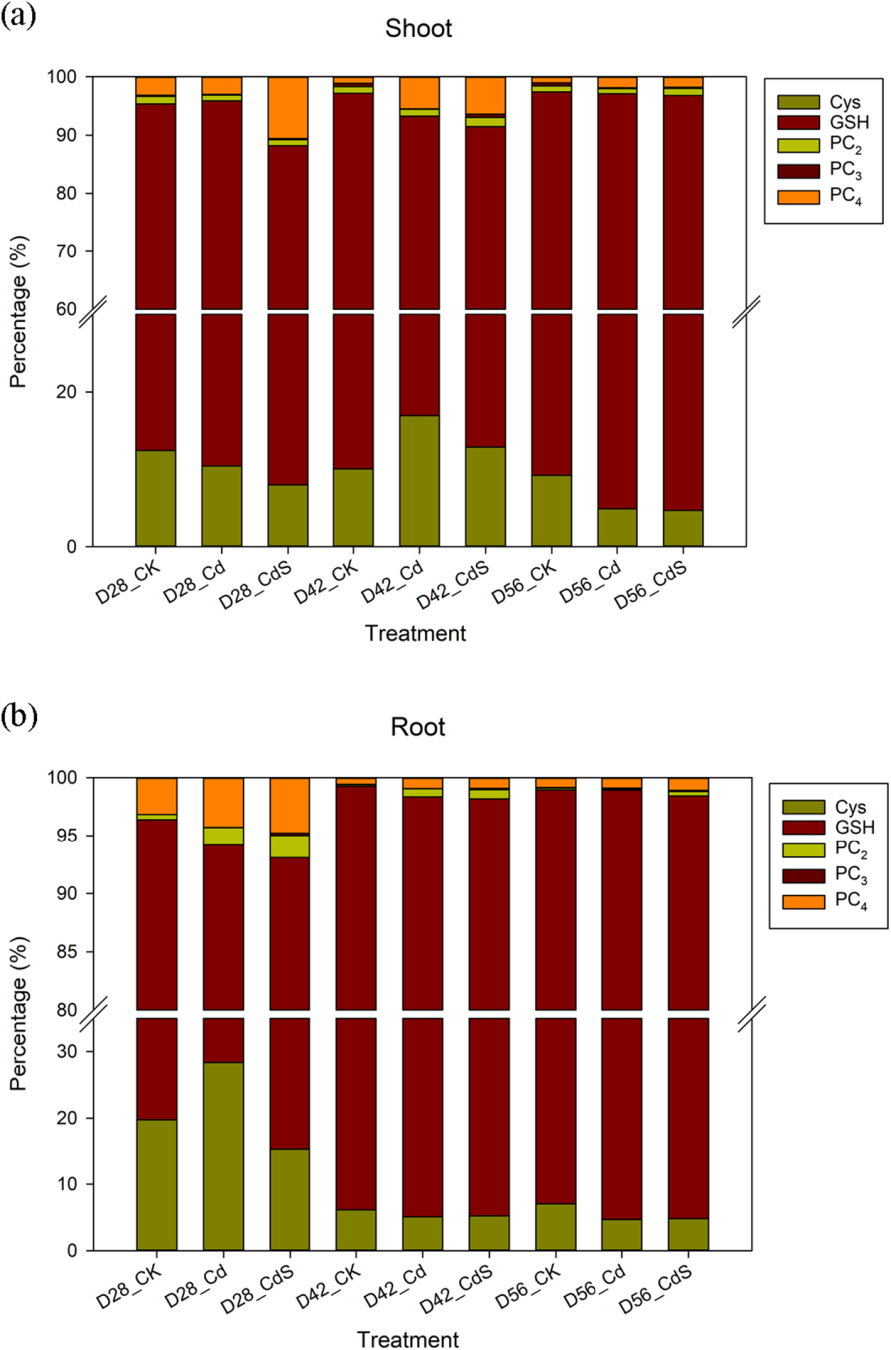
Percentage of different thiols compounds in shoots (a) and roots (b) after the hydroponic experiment under different treatments. CK: 0 μM CdCl_2_ + 0 mM Na_2_SO_4_; Cd: 40 μM CdCl_2_ + 0 mM Na_2_SO_4_; CdS: 40 μM CdCl_2_ + 4 mM Na_2_SO_4_; D28: harvest at 28 days; D42: harvest at 42 days; D56: harvest at 56 days. Cys: cysteine; GSH: glutathione; PC_2_: phytochelatin 2; PC_3_: phytochelatin 3; PC_4_: phytochelatin 4.

**Table 1:** Percentage of Phytochelatin and Phytochelatin–Cd Complexes of the Lettuce in Shoots after the Hydroponic Experiment under Different Treatments^a^

	PC_2_	PC_3_	PC_4_	PC_2_–Cd	PC_3_–Cd	PC_4_–Cd	PC_S_	PC_S_-Cd
treatment	------------------------------------------------------%-----------------------------------------------------------							
D28_Cd	9.86	1.93	66.78	14.37	1.19	5.87	78.57	21.43
D28_CdS	3.61	0.66	69.70	5.04	0.92	20.06	73.97	26.03
D42_Cd	9.06	0.86	62.77	8.29	0.43	18.59	72.69	27.31
D42_CdS	5.99	3.70	44.65	12.41	2.59	60.67	54.34	75.66
D56_Cd	19.01	2.61	18.21	11.12	2.43	46.63	39.82	60.18
D56_CdS	22.36	1.77	0.23	15.54	3.58	66.52	24.36	85.64

a CK: 0 μM CdCl_2_ + 0 mM Na_2_SO_4_; Cd: 40 μM CdCl_2_ + 0 mM Na_2_SO_4_; CdS: 40 μM CdCl_2_ + 4 mM Na_2_SO_4_; D28: harvest at 28 days; D42: harvest at 42 days; D56: harvest at 56 days; PC_2_: phytochelatin 2; PC_3_: phytochelatin 3; PC_4_: phytochelatin 4; PCs: phytochelatins; PC_2_–Cd: phytochelatin 2-Cd complex; PC_3_–Cd: phytochelatin 3-Cd complex; PC_4_–Cd: phytochelatin 4-Cd complex; PCs–Cd: phytochelatins–Cd complex.

**Table 2:** Percentage of Phytochelatin and Phytochelatin–Cd Complexes of the Lettuce in the Roots after the Hydroponic Experiment under Different Treatments^a^

	PC_2_	PC_3_	PC_4_	PC_2_–Cd	PC_3_–Cd	PC_4_–Cd	PC_S_	PC_S_-Cd
treatment	------------------------------------------------------%-----------------------------------------------------------							
D28_Cd	20.78	0.10	46.77	5.20	0.21	26.94	67.65	32.35
D28_CdS	11.18	1.32	37.49	16.76	1.56	31.69	49.99	50.01
D42_Cd	19.97	0.88	17.90	5.94	0.99	38.32	38.75	45.25
D42_CdS	13.99	2.52	0.49	28.98	3.69	50.33	17.00	83.00
D56_Cd	7.77	1.01	4.00	2.85	3.82	80.55	12.78	87.22
D56_CdS	7.33	0.67	1.31	16.38	6.19	68.12	9.31	90.69

a CK: 0 μM CdCl_2_ + 0 mM Na_2_SO_4_; Cd: 40 μM CdCl_2_ + 0 mM Na_2_SO_4_; CdS: 40 μM CdCl_2_ + 4 mM Na_2_SO_4_; D28: harvest at 28 days; D42: harvest at 42 days; D56: harvest at 56 days; PC_2_: phytochelatin 2; PC_3_: phytochelatin 3; PC_4_: phytochelatin 4; PCs: phytochelatins; PC_2_–Cd: phytochelatin 2-Cd complex; PC_3_–Cd: phytochelatin 3-Cd complex; PC_4_–Cd: phytochelatin 4-Cd complex; PCs–Cd: phytochelatins–Cd complex.

### 3.5 AsA–GSH Cycle

As illustrated in Figure 7, exposure to Cd led to elevated contents of AsA and GSH in both shoots and roots when compared to the control group. Furthermore, the addition of S to Cd-treated plants resulted in a significant increase (*p* < 0.05) in AsA and GSH contents relative to Cd treatment alone. In Figure 8, it is evident that the activities of APX, GR, and DHAR were markedly enhanced in both tissues under Cd stress compared to the control treatment. These enzyme activities were further amplified when S was coapplied with Cd. Notably, the peak content of both AsA and GSH, along with the highest enzyme activities of APX, GR, and DHAR, was observed on D42.

**Figure 7:**
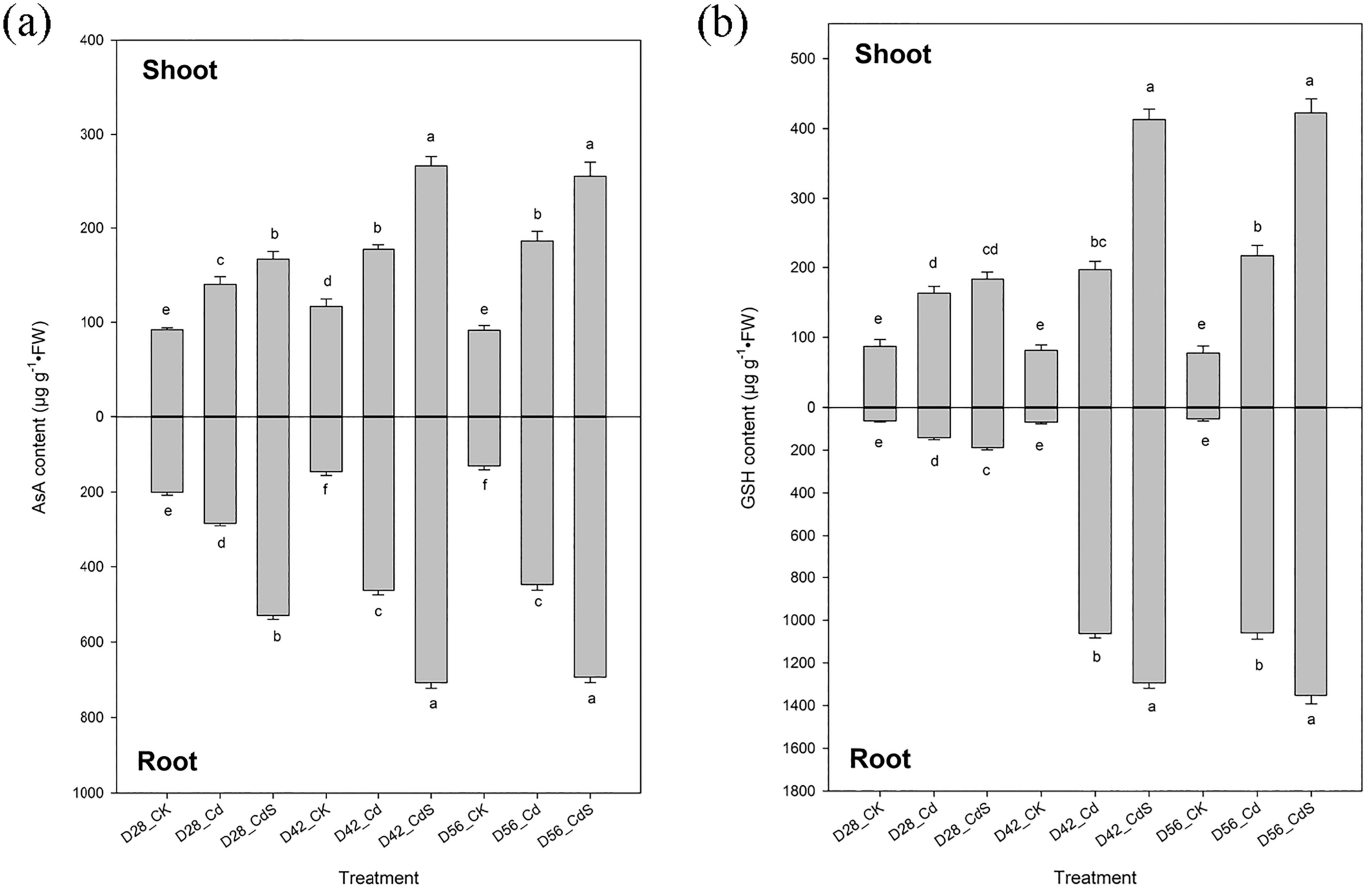
AsA (a) and GSH (b) concentrations of lettuce after the hydroponic experiment under different treatments. Small letter indicates statistical differences among treatments (LSD test, *p* < 0.05). CK: 0 μM CdCl_2_ + 0 mM Na_2_SO_4_; Cd: 40 μM CdCl_2_ + 0 mM Na_2_SO_4_; CdS: 40 μM CdCl_2_ + 4 mM Na_2_SO_4_; D28: harvest at 28 days; D42: harvest at 42 days; D56: harvest at 56 days.

**Figure 8:**
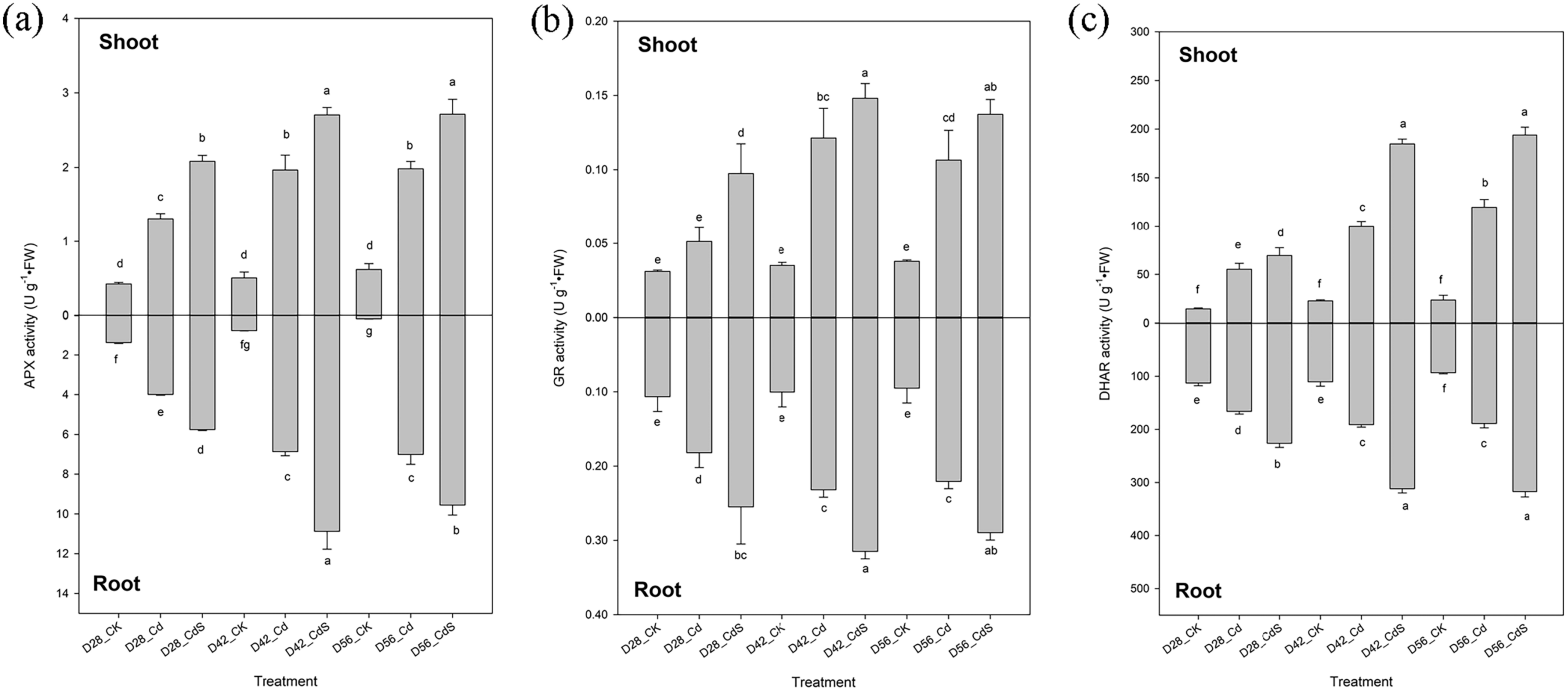
Activity of APX (a), GR (b), and DHAR (c) of lettuce after the hydroponic experiment under different treatments. Small letter indicates statistical differences among treatments (LSD test, *p* < 0.05). CK: 0 μM CdCl_2_ + 0 mM Na_2_SO_4_; Cd: 40 μM CdCl_2_ + 0 mM Na_2_SO_4_; CdS: 40 μM CdCl_2_ + 4 mM Na_2_SO_4_; D28: harvest at 28 days; D42: harvest at 42 days; D56: harvest at 56 days.

## 4 Discussion

### 4.1 Role of Sulfur in Promoting Plant Growth under Cd Stress

Cd toxicity reduces chlorophyll content, disrupts photosystem efficiency, and suppresses biomass accumulation.^39,40^ S supplementation can mitigate these effects by enhancing plant growth under Cd stress, as evidenced by improved rice yield^41^ and increased shoot height, root elongation, and fresh biomass in pakchoi.^42^ In our study, lettuce exposed to 40 μM Cd exhibited inhibited growth compared to the controls; however, S addition alleviated these effects, increasing shoot and root lengths, dry weight, and SPAD readings. These results indicate that S maintains photosynthetic efficiency and overall plant vitality, thereby partially offsetting Cd-induced growth inhibition.

### 4.2 Sulfur Reduced Cd Accumulation and Translocation

Within plants, thiols—including GSH and PCs—act as metal chelators, detoxifying harmful ions such as Cd.^43^ S supplementation promotes thiol biosynthesis, limits Cd uptake, and enhances biomass accumulation.^44,45^ For example, S increased GSH and PCs in rice under Cd stress and reduced Cd accumulation in wheat and lettuce roots under low Cd exposure (0.02–0.04 mM) through elevated thiol production.^45^ In our study, S application similarly increased internal S and thiol contents, decreased Cd accumulation and translocation, and enhanced the proportion of PCs within the thiol pool. PCs, synthesized nonribosomally from GSH via phytochelatin synthases^46^ and typically represented by (γ–Glu–Cys)­n–Gly (*n* = 2–7), bind Cd­(II) more strongly than GSH, as confirmed by competition assays^47^ and potentiometric/spectroscopic studies showing increasing Cd affinity from GSH to PC_4_ (log K^7.4^ GSH = 5.93; log K^7.4^ PC_4_ = 13.39).^48^ PCs–Cd complexes are primarily sequestered in vacuoles, with over 97% of Cd in tolerant plants stored in this form,^49^ and ESI–MS/MS studies have characterized CdPC_3_ and CdPC_4_ in *Brassica chinensis*.^32^ Using SEC–HPLC–UV–Q-TOF, we detected [CdPC_2_+ H]^+^ (*m*/*z* = 652.0295), [CdPC_3_+ H]^+^ (*m*/*z* = 884.0815), and [CdPC_4_+ H]^+^ (*m*/*z* = 1116.1345) in lettuce under Cd stress, confirming in vivo complex formation. The proportion of PC_S_–Cd complexes increased over time, reaching 60.18% in shoots and 87.22% in roots by D56, consistent with reports in hyperaccumulator plants.^50,51^ The enhanced proportion of PC_4_ likely contributed to stronger metal-binding capacity, as the number of thiol groups positively correlates with chelation potential.^52^

### 4.3 Cd Sequestration into Vacuoles Enhanced by Sulfur

After chelation, PCs–Cd complexes are compartmentalized into vacuoles, while cell wall immobilization provides an additional detoxification strategy.^53^ Cd bound to cell wall components is prevented from entering the cytoplasm, reducing translocation to shoots and protecting sensitive organelles, as observed in rice and water spinach.^54,55^ In our study, Cd was mainly vacuolar, with S supplementation further increasing vacuolar Cd on D28 and D42. By D56, more Cd was associated with the cell wall, likely due to the saturation of PC–Cd complexes or progressive cell wall thickening, which enhances binding capacity. S may also promote lignin accumulation, further stabilizing Cd in the cell wall.^56,57^ Together, vacuolar sequestration and cell wall immobilization effectively retain Cd in roots and minimize shoot translocation, consistent with the observed reduction in Cd TF.^58,59^

### 4.4 Sulfur Enhanced the AsA–GSH Cycle

The AsA–GSH cycle detoxifies H_2_O_2_ and generates antioxidants, including AsA and GSH, which further scavenge ROS.^60^ Cd accumulation elevates ROS and lipid peroxidation, exacerbating oxidative stress.^61^ S application enhanced the AsA–GSH cycle in Cd-stressed lettuce, consistent with reports that S supplementation increases Cys, GSH, and AsA contents and activates antioxidant enzymes and AsA–GSH-related genes in pakchoi.^62^ Cd accumulation peaked around D42 and stabilized or slightly declined by D56, reflecting its nonessential nature and uptake via transporters shared with essential metals such as zinc (Zn^2+^), iron (Fe^2+^), calcium (Ca^2+^), and manganese (Mn^2+^).^63^ Plant growth was most vigorous up to D42, then plateaued, potentially due to physiological maturation or reduced nutrient uptake; decreased transpiration and root activity may also contribute.^64,65^ Accordingly, S and thiol levels and AsA–GSH activity plateaued or slightly declined by D56, suggesting diminished detoxification demand as Cd stress lessened.

In conclusion, our results suggest that S may enhance lettuce tolerance to Cd stress through several potentially interconnected mechanisms based on observational evidence: (i) promoting S assimilation and thiol (GSH and PC) production, (ii) facilitating PC_S_–Cd complex formation and vacuolar sequestration, (iii) stabilizing Cd in cell walls, and (iv) reinforcing antioxidant defenses via the AsA–GSH cycle. Subcellular distribution analyses indicated that S increased Cd sequestration in the cell wall and vacuolar compartment, and may limit Cd translocation from roots to shoots and reduce the risk of human intake. Although the hydroponic Cd concentrations were higher than environmentally relevant levels and preclude direct risk assessment, these findings provide a mechanistic foundation for future soil-based or field studies to further validate these responses. Such studies could also assess the potential of S fertilization for mitigating Cd accumulation in edible tissues, thereby contributing to safer and more sustainable cultivation practices.

## Supplementary Material



## Notes

Additional data and figures that complement the findings presented in the main text. Figure S1 shows the Cd translocation factor after the hydroponic experiment under different treatments. Figures S2–S4 present Q-TOF spectra of the in vitro synthesized PC2–Cd, PC3–Cd, and PC4–Cd complexes, respectively. Figure S5 provides a schematic diagram of the experimental setup. Figure S6 illustrates the changes in Cd subcellular distribution under Cd treatment (a) and Cd+S treatment (b) across different time points. Figure S7 proposes a mechanism of sulfur-mediated Cd detoxification in lettuce. Collectively, these materials provide additional evidence supporting the proposed detoxification pathways and experimental interpretations (PDF)
