# Supplementary material for: Sulfur Mitigates Cadmium Toxicity in Lettuce via Phytochelatins and the AsA–GSH Cycle
Source: J Agric Food Chem. 2025 Oct 13;73(42):26658–68. doi: 10.1021/acs.jafc.5c09833 (PMC12550847; doi:10.1021/acs.jafc.5c09833)
Supplement: Supplementary file 1 [file jf5c09833_si_001.pdf]

Sulfur Mitigates Cadmium Toxicity in Lettuce via Phytochelatins and the AsA–GSH Cycle

Kuei–San Chen[a] and Hung–Yu Lai[a]*

[a] Department of Soil and Environmental Sciences, National Chung Hsing University, Taichung 402202, Taiwan

[*] Correspondence: soil.lai@nchu.edu.tw

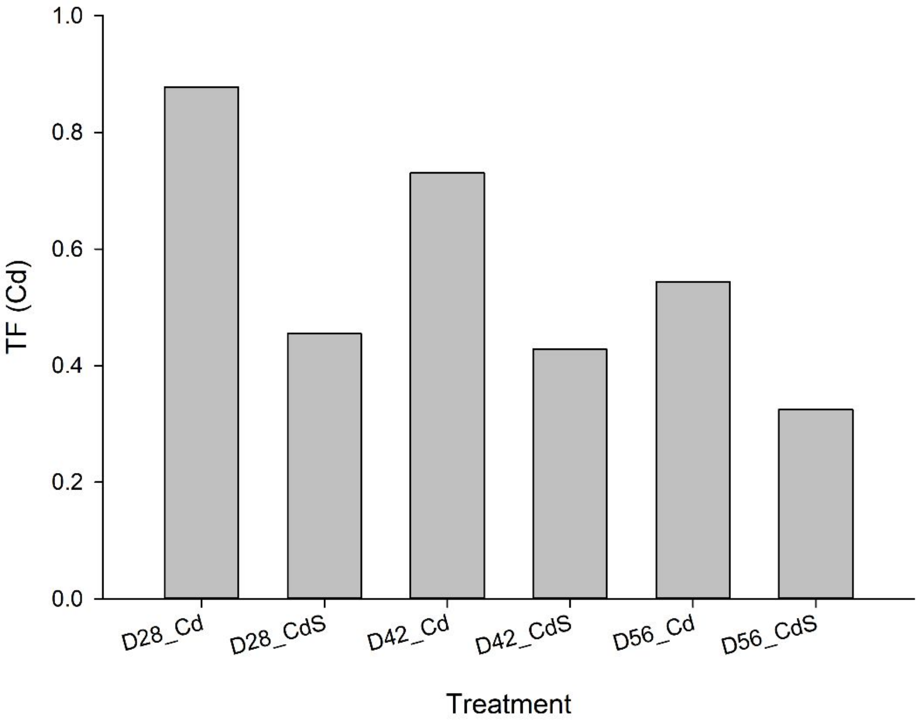

Figure S1. Translocation factor of Cd after the hydroponic experiment under different treatments. CK: 0 μM $CdCl_2$ + 0 mM Na_2SO_4; Cd: 40 μM $CdCl_2$ + 0 mM Na_2SO_4; CdS: 40 μM $CdCl_2$ + 4 mM Na_2SO_4; D28: harvest at 28 days; D42: harvest at 42 days; D56: harvest at 56 days

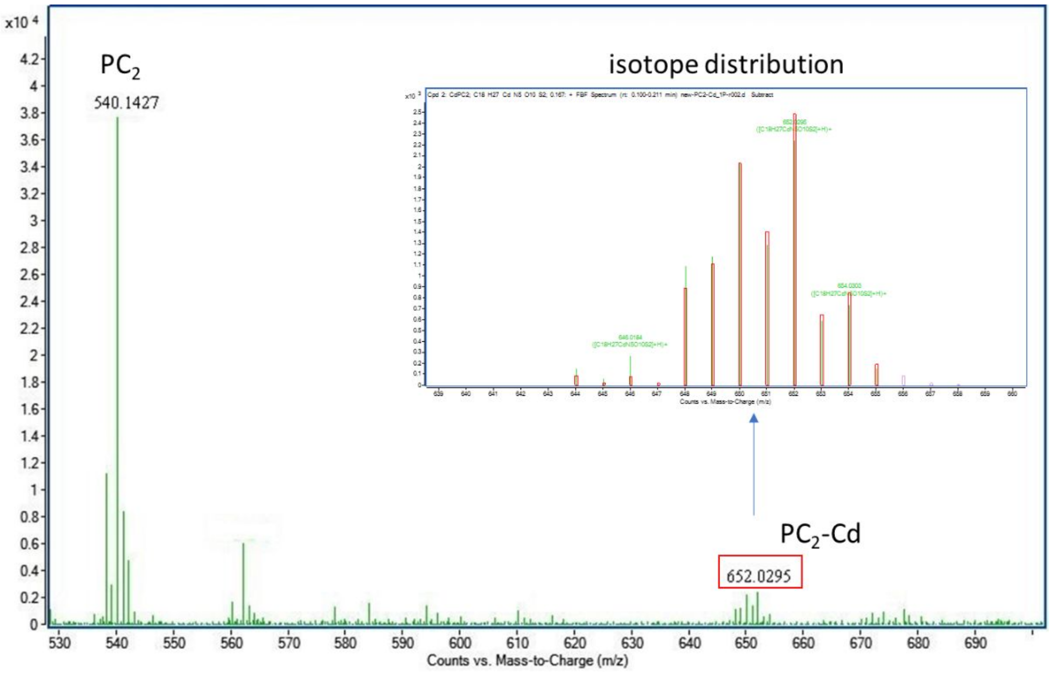

Figure S2. Q-TOF spectra of the in vitro synthesized PC2-Cd.

PC_2: phytochelatin 2; PC_2-Cd: phytochelatin 2-Cd complex

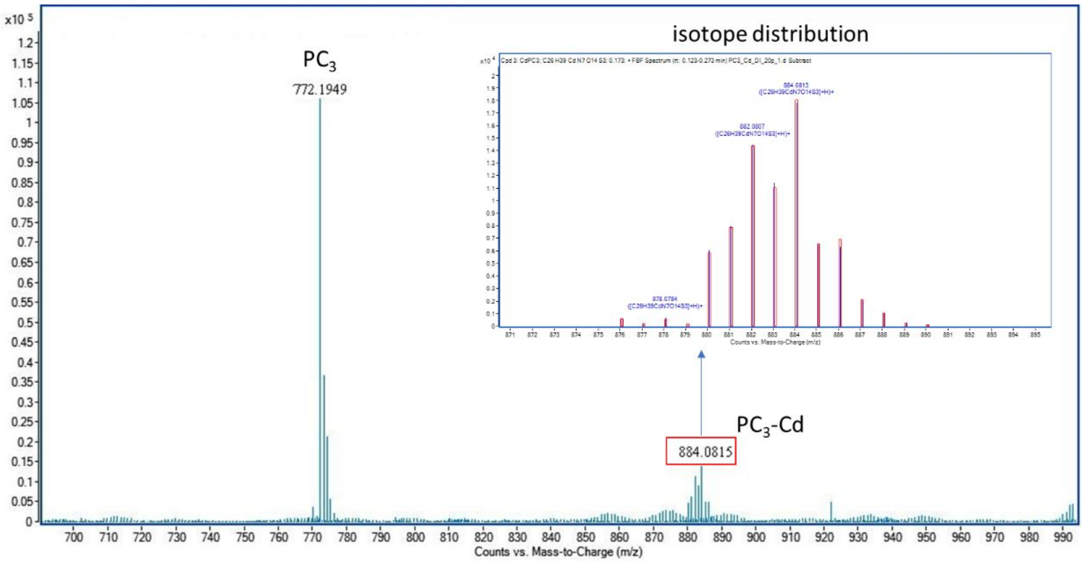

Figure S3. Q-TOF spectra of the in vitro synthesized PC_3-Cd.

PC_3: phytochelatin 3; PC_3-Cd: phytochelatin 3-Cd complex

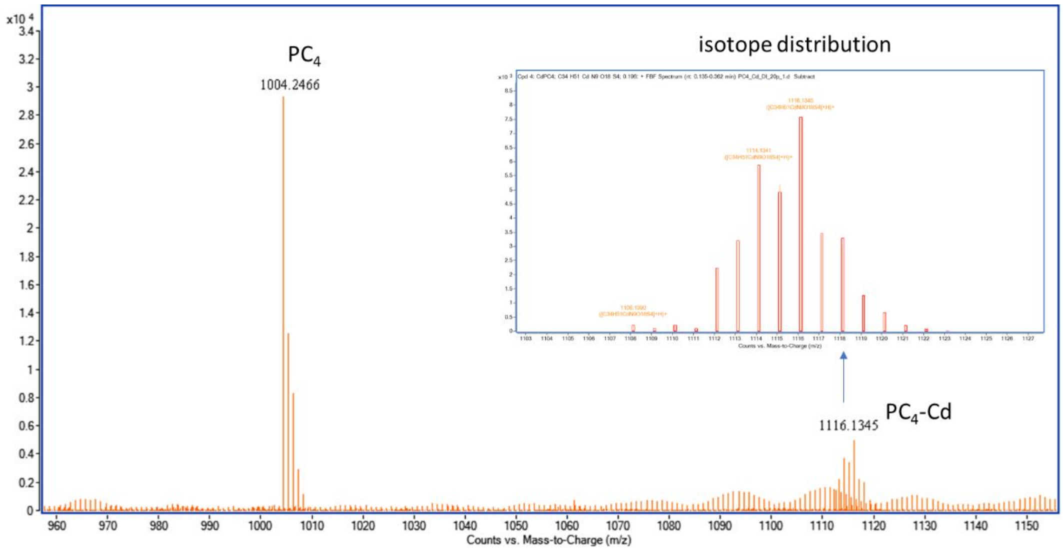

Figure S4. Q-TOF spectra of the in vitro synthesized PC_4-Cd.

PC_4: phytochelatin 4; PC_4-Cd: phytochelatin 4-Cd complex

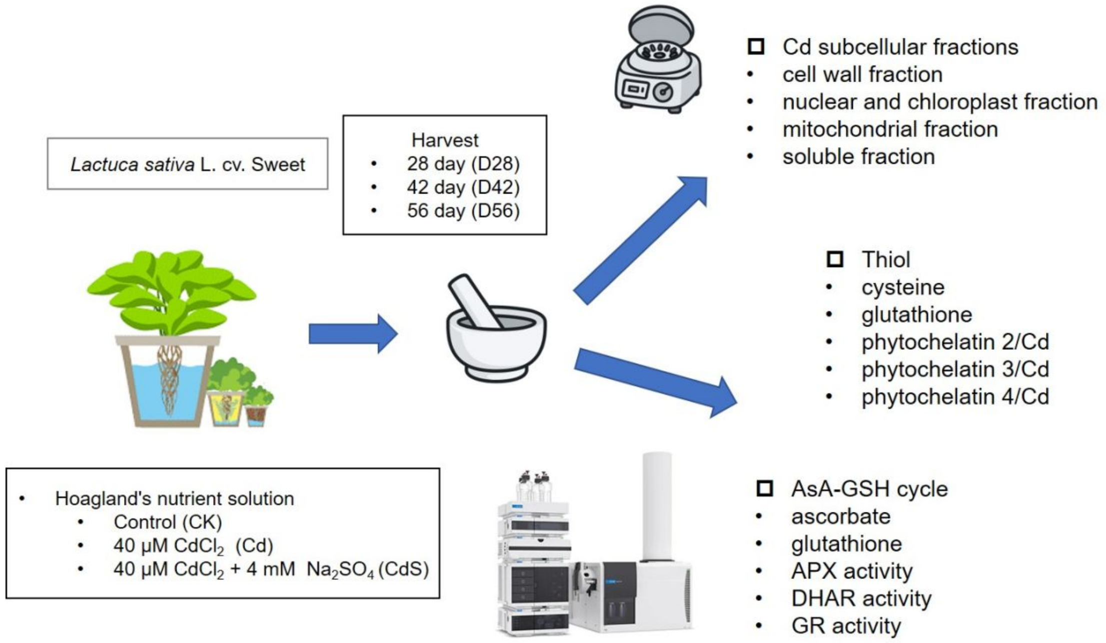

Figure S5. Schematic diagram of the experiment.

(a)

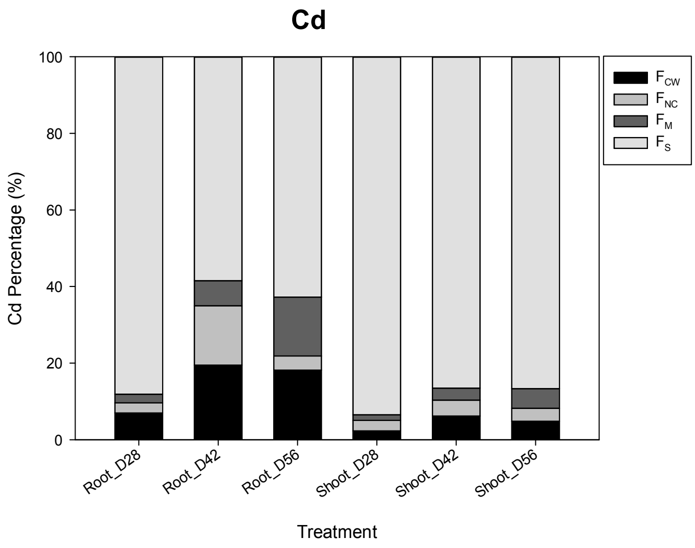

(b)

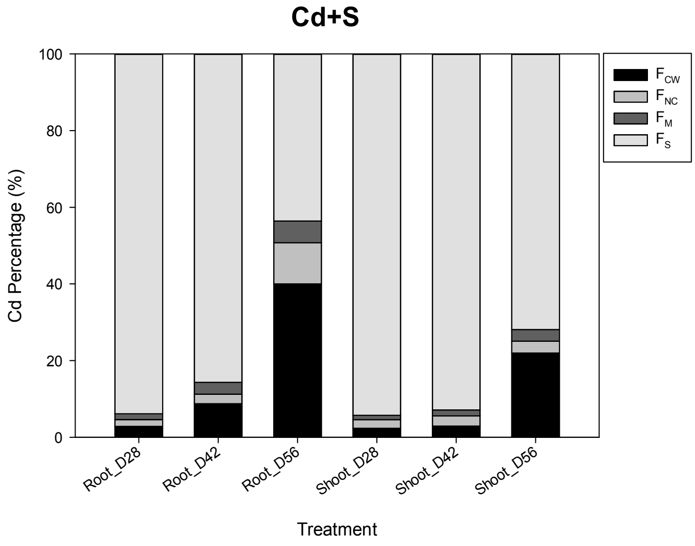

Figure S6. Changes in Cd subcellular distribution under Cd treatment (a) and Cd+S treatment (b) across different time points.

CK: 0 μM $CdCl_2$ + 0 mM Na_2SO_4; Cd: 40 μM $CdCl_2$ + 0 mM Na_2SO_4; CdS: 40 μM $CdCl_2$ + 4 mM Na_2SO_4; D28: harvest at 28 days; D42: harvest at 42 days; D56: harvest at 56 days.

F_{CW}: cell wall fraction; F_{NC}: nuclear and chloroplast fraction; F_M: mitochondrial fraction; F_S: soluble fraction

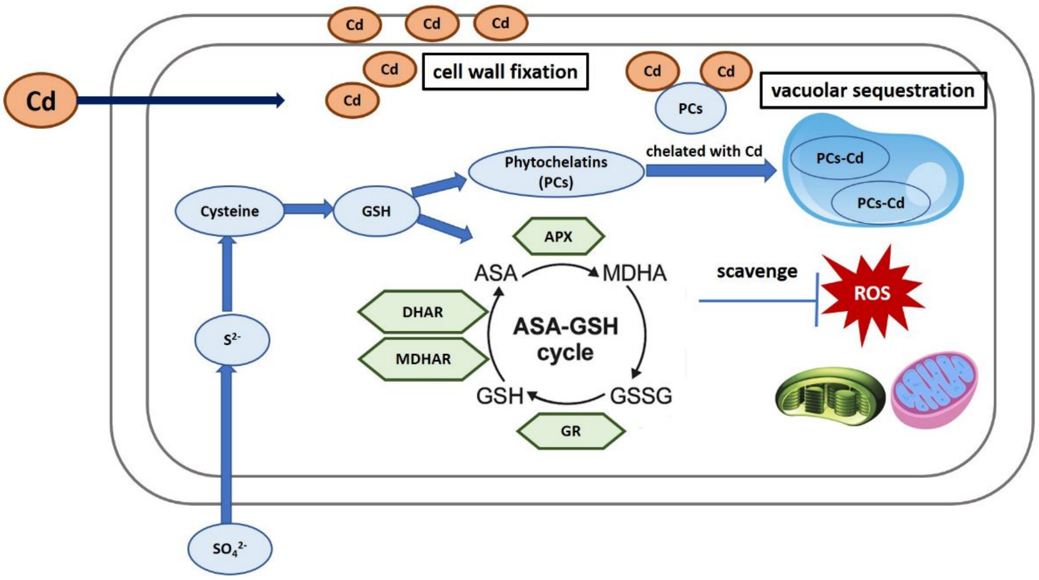

Figure S7. Proposed mechanism of S-mediated Cd detoxification in lettuce.
